# Automatic recognition and measurement of anatomical structures associated with the elevation of the maxillary sinus floor by deep learning on cone-beam computed tomographic scans

**DOI:** 10.1186/s12903-025-07609-4

**Published:** 2026-01-05

**Authors:** Bin Xuan, Qiang Ding, Weili Wang, Zhuojue Liu, Yajie Wang, Feifei Zuo, Jinlei Yin, Pan Yang

**Affiliations:** 1 Department of Stomatology, Aerospace Center Hospital, No.15 Yu-quan Road, Haidian District, Beijing, 100049 China; 2 Department of Logistics Support, Aerospace Center Hospital, No.15 Yu-quan Road, Haidian District, Beijing, 100049 China; 3 LargeV Instrument Corporation Limited, Room B701, Building B-2, No. 66 Xixiaokou Road, Haidian District, Beijing, 100192 P.R. China; 4 Department of Oral and Maxillofacial Radiology, Beijing Stomatological Hospital, School of Stomatology, Capital Medical University, Beijing, 100070 China

**Keywords:** Maxillary sinus floor elevation, Deep learning, Cone beam computed tomography, Multitasking, Dental implant

## Abstract

**Background:**

The purpose of this study is to develop a deep learning model that can identify the maxillary sinus, posterior superior alveolar artery(PSAA), and alveolar ridge, and evaluate its diagnostic performance. Based on this, relevant parameters for preoperative design of maxillary sinus elevation can be measured to achieve intelligent preoperative design for maxillary posterior tooth implantation surgery.

**Methods:**

A total of 2400 CBCT slices from patients with maxillary posterior tooth loss was selected as the initial dataset. Anatomical structure annotation and enhanced YOLOv11 architecture were used for model training to achieve segmentation of maxillary sinus, PSAA, and alveolar ridge. Intersection over union (IoU), average precision (AP), average recall (AR) and the Euclidean distance were used to evaluate the accuracy of structure segmentation. On the basis of the segmentation of the three important anatomical structures mentioned above, five anatomical parameters (A1-A5) related to maxillary posterior tooth implantation were set, and their errors were statistically analyzed.

**Results:**

The median IoU for maxillary sinus segmentation was 0.945 (IQR: 0.934–0.951, 95%CI: 0.935–0.941), while the median IoU for PSAA segmentation was 0.991 (IQR: 0.982–1.000, 95%CI: 0.948–0.974). The model achieved an average precision of 0.902 ± 0.023 and a recall of 0.937 ± 0.024 for PSAA segmentation. For alveolar crest localization, the mean Euclidean distance errors between predicted and ground-truth landmarks were 0.50 ± 0.31 mm and 0.38 ± 0.24 mm for the two key points, respectively. 95% of AI prediction errors for A1-A4 were within 1 mm, while 95% of AI prediction errors for A5 were within 10 mm^2^.

**Conclusions:**

The enhanced YOLOv11 framework reliably and autonomously identifies critical anatomical structures for maxillary sinus elevation including the maxillary sinus, PSAA, and maxillary alveolar crest in CBCT images. This model enables the acquisition of reliable clinical parameters, demonstrating its potential for future intelligent assisted preoperative evaluation and design of maxillary posterior dental implant surgery.

**Supplementary Information:**

The online version contains supplementary material available at 10.1186/s12903-025-07609-4.

## Background

Dental implants have become the treatment of choice for clinicians and patients seeking to restore missing teeth. However, insufficient vertical bone height for implant placement frequently occurs in the posterior maxilla due to long-term tooth loss or pneumatization of the maxillary sinus. To address this deficiency, maxillary sinus floor elevation is routinely performed, employing either a transalveolar or lateral window (fenestration) approach. If the residual bone height is over 5 mm, the transalveolar approach is preferable because it is less invasive and often used in conjunction with simultaneous bone grafting. When the available bone height falls below 5 mm, the lateral window approach is generally preferred to achieve sufficient augmentation [1]. The vertical bone height between the alveolar crest and the sinus floor is a critical parameter for pre-operative assessment.

Beyond vertical bone height, several anatomical parameters must be evaluated preoperatively to minimize complications and optimize surgical outcomes. These include the shape of the sinus, the existence and orientation of septa, the thickness of the Schneiderian membrane, and the route of intrasinus arteries, particularly the posterior superior alveolar artery (PSAA).

Maxillary sinus elevation surgery with lateral fenestration carries inherent risks, notably potential damage to PSAA during bony window preparation [2]. Accurate localization of PSAA is therefore paramount to prevent complications such as intraoperative bleeding, which can be challenging to control, especially when the artery is embedded in bone, and may lead to critical issues like sinus membrane perforation. Consequently, precise pre-operative assessment of PSAA location using imaging modalities like CBCT is essential for safe surgical planning and bony window design [3]. While parameters for the bony window are typically guided by anatomical landmarks (e.g., distance from sinus floor, anterior wall, alveolar crest), the variable course of the PSAA requires specific attention during the design of the superior border of the window [4].

Traditional two-dimensional imaging can distort such complex three-dimensional (3D) structures, underscoring the necessity of 3D imaging for comprehensive assessment [5]. Conebeam computed tomography (CBCT) has become the preferred method due to its superior spatial resolution, lower radiation exposure, faster scan times, and cheaper cost when compared to standard CT [6]. At the same time, convolutional neural networks (CNNs), a type of deep learning algorithm, have shown an outstanding capacity for autonomously identifying and categorizing image features. Although CNN-based systems require extensive annotated datasets to achieve optimal diagnostic accuracy, recent studies have shown that AI performance in lesion detection can rival that of experienced clinicians. Consequently, AI applications in CBCT imaging diagnosis have gained considerable traction [7].

In this study, we develop and validate a CBCT-based deep learning framework to automatically identify and quantify key anatomical structures relevant to maxillary sinus floor elevation—including sinus morphology, PSAA position, and alveolar crest level. To our knowledge, this study was the first to achieve the simultaneous segmentation of important anatomical structures such as maxillary sinus, PSAA, and alveolar ridge using deep learning models. Based on this, relevant clinical anatomical parameters were expanded to intelligently assist in preoperative evaluation and surgical design. By streamlining preoperative imaging analysis, our approach aims to enhance clinical decision-making and improve surgical planning.

## Methods

### Ethical approval and Study Participants

The Department of Stomatology at Aerospace Center Hospital provided the anonymized CBCT data used in this retrospective analysis. The image screening rules for this study are as follows:

Inclusion criteria: 1, Patients with maxillary posterior tooth loss. 2, The maxillary sinus is well defined. 3, Patients without history of maxillary sinus trauma and surgery.

Exclusion criteria: 1, Bilateral maxillary sinus dysplasia or lesions. 2, Images in which the maxillary sinus boundary cannot be clearly observed due to various artifacts (metal artifacts, motion artifacts, etc.).

After the above screening, a total of 667 patients’ CBCT images were included in this study for model training and testing. Records from 667 patients who presented between January 2022 and April 2023 for planned dental implant placement in the maxillary posterior region were considered for inclusion. The patients’ ages ranged from 30 to 72 years old, the male to female ratio was 334 to 333. This study was conducted in accordance with the ethical principles of the World Medical Association’s Declaration of Helsinki. The Ethics Committee of the Aerospace Center Hospital granted ethical approval for the research procedure (Ethics No. 2023/03501).

### Cornal slices acquisition and preprocessing

All CBCT scans were conducted using the same CBCT device (KaVo 3D eXam, KaVo Dental, Biberach, Germany) in order to maintain uniformity. The acquisition parameters were standardized as follows: a field of view (FOV) of 16 cm (diameter)×19 cm (height), 360°rotation, isotropic voxel size of 0.3 mm, X-ray tube current ranging from 1 mA to 11 mA, tube voltage of 110 kVp, and scan time of 3.6 s. All patients’ CBCT data were downloaded and stored in DICOM format. The DICOM files were processed using SmartVPro software (version 2.0, open source). Two-dimensional (2D) slices of the maxillary sinus were taken manually using the software’s coronal window. The parameters of coronal window were: inter-slice distance of 1 mm, slice width of 70 mm, and slice thickness of 0.9 mm. All slices were completed by a radiologist with 8 years of experience in oral and maxillofacial radiology. We manually selected 2400 2D coronal slices as the final dataset which were stored and uploaded in JPEG format.

### Image annotation and ground truth generation

**Fig. 1:**
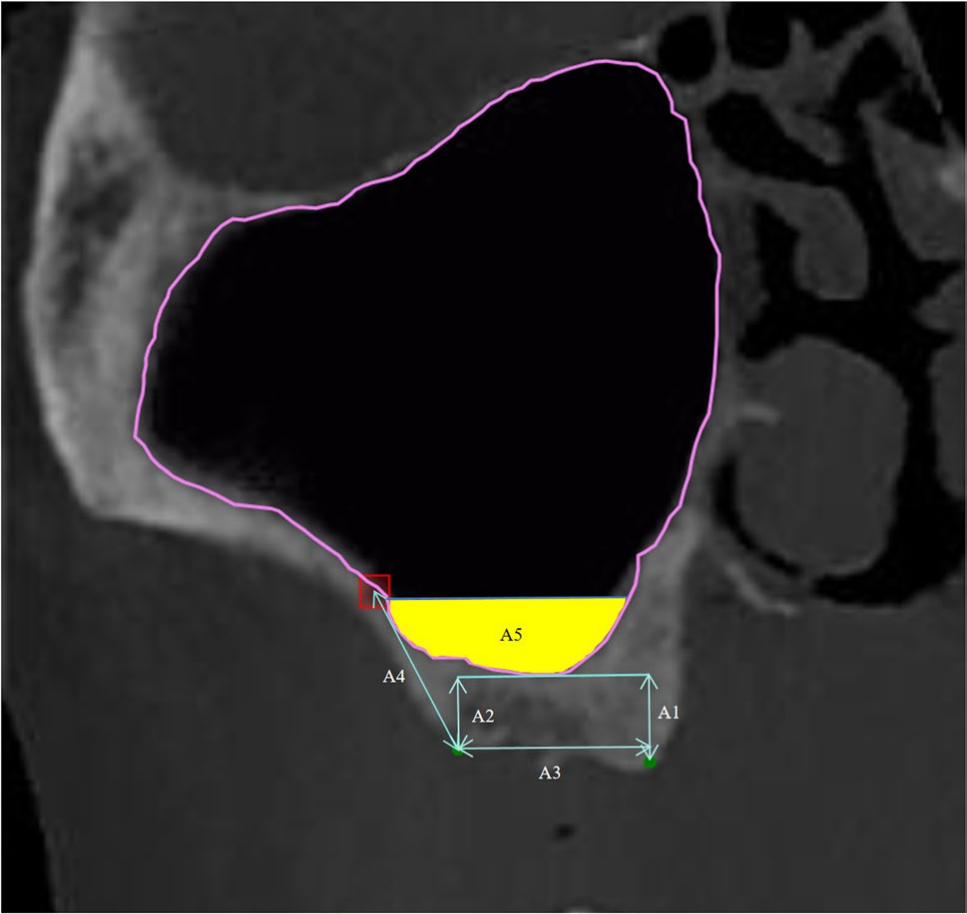
Manual labeling schematic and visualization of A1-A5 calculations. The boundary of the maxillary sinus is defined by the uneven contour lines in the labeled CBCT images. The red rectangles show where PSAA is, and the green dots show the buccal and palatal turning points of the alveolar crest in the posterior maxilla

The resulting 2400 selected coronal slices were uploaded to an online annotation platform (Label Studio, accessible via https://label.ai-align.cn/). In an 80:10:10 ratio, the dataset was split into a training set, a validation set, and a testing set. Ground-truth annotations were meticulously performed by two oral surgeons with over five years of clinical experience. Using polygonal marking tools, they delineated the outer boundaries of relevant maxillary sinus-related anatomical structures to create precise label masks for model training and evaluation (as illustrated in Fig. 1).

To assess annotation consistency, two experienced oral surgeons independently labeled the coronal slices. For maxillary sinus segmentation, the Intersection over Union (IoU) metric was calculated by comparing the overlap between the two annotations. A higher IoU indicates a greater degree of agreement. We selected 200 slices from the training set for consistency testing. The average IoU is greater than 0.9, indicating excellent consistency. For the 5 slices with an IoU lower than 0.9, another senior physician conducted an arbitration assessment to resolve disputes and obtain high-quality dataset annotations.

For PSAA and alveolar crest annotation, the L2 pixel distance (Euclidean distance) was used to evaluate the deviation between the center points of the rectangular annotations for the PSAA and the midpoints of the alveolar crest line. The observed differences were less than one pixel, indicating high precision and inter-observer agreement.

$$\mathrm L2=\sqrt{\left(x_2-x_1\right)^2+\left(y_2-y_1\right)^2}$$

**Fig. 2:**
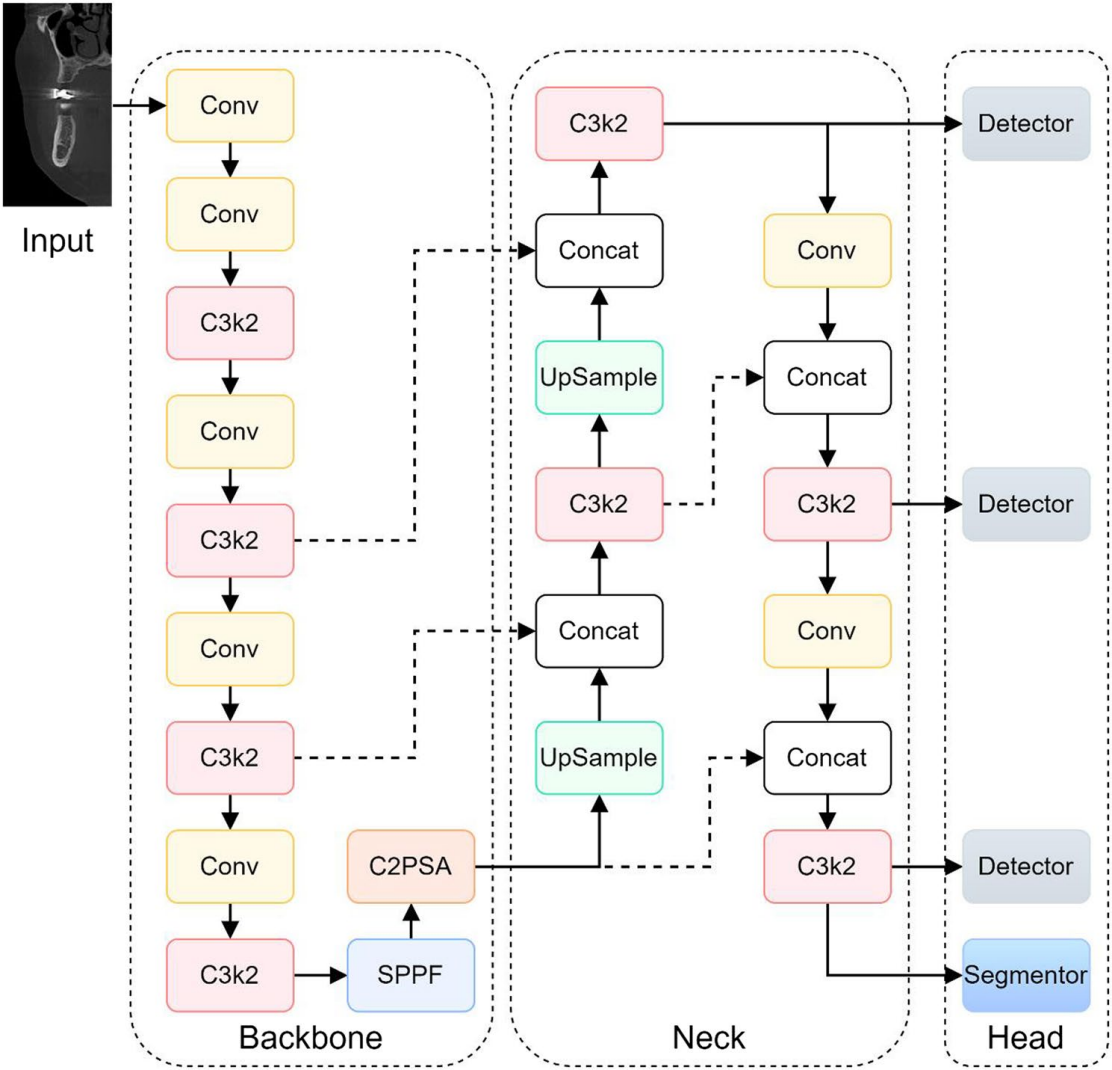
Overall Network Architecture Framework

This study proposes an integrated network based on an enhanced YOLOv11 framework, specifically engineered for the challenging tasks of maxillary sinus segmentation, PSAA detection, and alveolar ridge apex identification within complex medical imaging data, particularly those involving curved fragments. The entire design, as illustrated in Fig. 2, is made up of three main parts: the Head, Neck, and Backbone.

The Backbone module is primarily responsible for hierarchical feature extraction. It commences with five convolutional layers, where the initial layer down-samples the input using a 3 × 3 kernel and a stride of 2, effectively reducing spatial dimensions while increasing feature depth. The C3K2 Module incorporates an improved Cross-Stage Partial (CSP) bottleneck design. This design enhances computational efficiency by replacing larger convolutions with a sequence of two smaller ones and splitting the feature map into parallel paths—one processed through bottlenecks and the other bypassed—before merging. This strategy reduces computational load while improving feature representation. Subsequently, the Spatial Pyramid Pooling-Fast (SPPF) Module is employed to capture multi-scale features through max pooling with varying window sizes. This operation is crucial for robust object detection across different scales, enabling the network to effectively detect both small and large objects. Feature refinement is further enhanced by the Cross-Stage Partial Spatial Attention (CSPSA) Module, which directs focus to salient image regions via spatial pooling. Complementing this, Multiple Position-Sensitive Attention (PSA) Blocks are utilized to extract fine-grained self-attention features, thereby improving the detection accuracy for small or occluded objects and enhancing overall localization precision.

The Neck component is designed for the aggregation and refinement of multi-scale features extracted by the Backbone. It primarily consists of C3K2 blocks, concatenation layers, and upsampling layers. C3K2 blocks continue to process and transform the features efficiently. Upsampling layers increase the spatial resolution of lower-level feature maps to align with those from higher levels. Concatenation layers then fuse these features from different resolutions, integrating fine-grained spatial details with coarser contextual information. This multi-scale feature aggregation strategy is integral to enhancing the network’s capacity for accurate detection and segmentation of complex targets.

The Head component is specifically tailored to generate predictions for the three distinct tasks. For PSAA detection, a dedicated detection head outputs bounding boxes along with class confidence scores. Maxillary sinus segmentation is addressed using both a detection head to localize the sinus region and a segmentation head that generates precise pixel-wise masks within the predicted bounding boxes. For alveolar ridge apex identification, the head integrates object detection with keypoint localization: it predicts a bounding box identifying the apex region and simultaneously outputs the corresponding$\:({x}_{k},{y}_{k})$ keypoint coordinates. This combined approach, predicting a bounding box alongside the specific keypoint within it, is particularly beneficial for precise anatomical landmark localization.

**Fig. 3:**
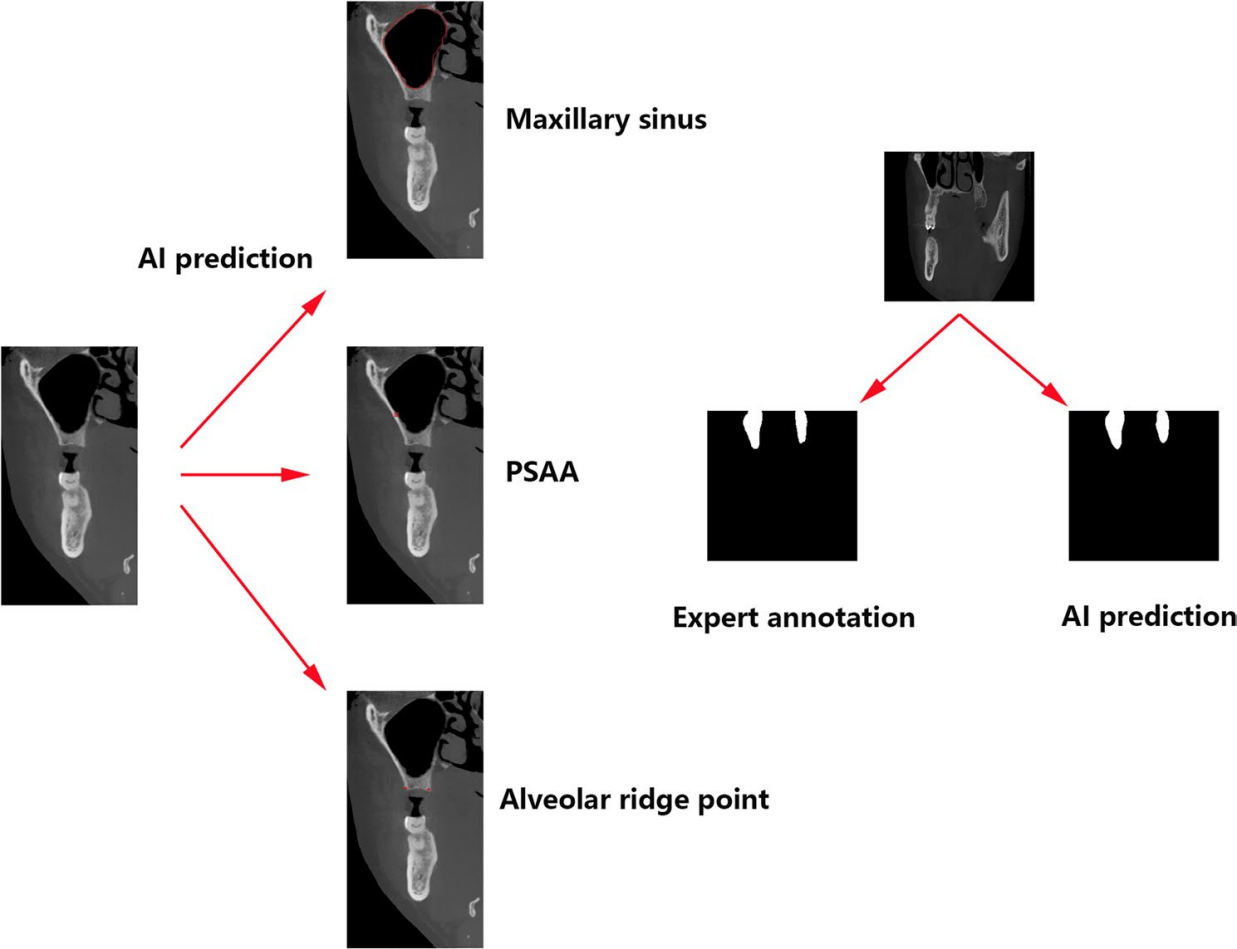
Visualization of segmentation for maxillary sinus, PSAA, and alveolar ridge points

This multi-task network design, which integrates detection, segmentation, and keypoint localization capabilities, provides a robust and versatile framework for complex medical image analysis, significantly enhancing the precision of anatomical feature identification essential for applications like maxillary sinus elevation planning (as shown in Fig. 3).

### Evaluation metrics on the test datase

For maxillary sinus segmentation and PSAA segmentation, the IoU metric is employed to evaluate the consistency of manual cross-annotation and assess the performance of AI predictions. The ratio of the area of overlap between two regions to the area of their union is what is meant by IoU, where A∩B denotes the intersection of region A and region B, and A∪B represents their union.

$$\:IOU=\:\frac{A\cap\:B}{A\cup\:B}$$

The consistency of manual cross-annotation and the effectiveness of AI predictions are assessed using the COCO object detection evaluation metrics, namely Average Precision (AP) and Average Recall (AR), for PSAA detection. Specifically:

AP@[IoU = 0.5] represents the average precision at a fixed IoU threshold of 0.5.

AR@[IoU = 0.5] represents the average recall at a fixed IoU threshold of 0.5.

$$\:Precision=\frac{TP}{TP+FP}$$

$$\:Recall=\frac{TP}{TP+FN}$$

The definitions of key terms are as follows:

- True Positive (TP): The number of correctly predicted bounding boxes where [IoU > threshold], with each ground truth (GT) matched to only one prediction.
- False Positive (FP): The number of redundant predicted bounding boxes for a single GT where [IoU < threshold] or multiple predictions are matched to the same GT.
- False Negative (FN): The number of ground truth instances that were not detected (missed detections).

For alveolar ridge point detection, the Euclidean distance is used as the evaluation metric. The model’s accuracy is quantified by calculating the error between the predicted point and the annotated point, while the consistency of manual annotations is evaluated by measuring the Euclidean distance between points marked by different annotators.

Given two points, point1$\:{(x}_{1}{y}_{1})$, point2$\:{(x}_{2}{y}_{2})$, the Euclidean distance is calculated using the formula:

$$\:d=\:\sqrt{{({x}_{2}-{x}_{1})}^{2}+{({y}_{2}-{y}_{1})}^{2}}$$

### Relevant calculations

As illustrated in Fig. 1, the semi-automatic calculation of preoperative surgical design data includes determining the width of the alveolar crest, represented by A3 the projection length of the line connecting the two green-marked points along the horizontal plane. This width is calculated by measuring the difference in the x-axis coordinates between two points on the alveolar crest, $\:{P}_{1}$ ($\:{P}_{1}x$, $\:{P}_{1}y$) and $\:{P}_{2}$($\:{P}_{2}x$, $\:{P}_{2}y$).

$$\:A3=|{P}_{1}x,-{P}_{2}x|$$

The perpendicular line from the green-marked points on the alveolar crest to horizontal line of the lowest point of the maxillary sinus (A1 and A2) represents the distance between the maxillary sinus floor and the alveolar crest. The specific calculation involves the difference in the y-axis distance between the lowest point of the maxillary sinus, $\:{P}_{lowest}$($\:{P}_{lowest}x$, $\:{P}_{lowest}y$), and the two points on the alveolar crest, $\:{P}_{1}$ ($\:{P}_{1}x$, $\:{P}_{1}y$) and $\:{P}_{2}$($\:{P}_{2}x$, $\:{P}_{2}y$), calculated as the y-axis difference between $\:{P}_{lowest}$ and $\:{P}_{1}$​ and $\:{P}_{2}$ respectively.

$$\:A1=|{P}_{lowest}y,-{P}_{1}y|$$

$$\:A2=|{P}_{lowest}y,-{P}_{2}y|$$

The distance from the center point of the PSAA rectangular frame to the buccal marked point on the alveolar crest is denoted as A4. The specific calculation involves determining the distance difference between the center point of the arterial rectangular frame $\:{P}_{center}$($\:{P}_{center}x$, $\:{P}_{center}y$)and the buccal marked point of the alveolar crest $\:{P}_{b}$ ($\:{P}_{b}x$, $\:{P}_{b}y$).

$$\:A4=\:\sqrt{{({P}_{center}x-{P}_{b}x)}^{2}+{({P}_{center}y-{P}_{b}y)}^{2}}$$

The conventional length of the implant is 10 mm. When the remaining height of the alveolar bone is less than 10 mm, maxillary sinus floor elevation and bone grafting are required. The length of the implant in the maxillary sinus is evaluated by subtracting the remaining alveolar bone height from a fixed distance of 10 mm, in order to assess the subsequent bone graft volume. A 10 mm vertical line is drawn from the midpoint of the line connecting the two green-marked points on the alveolar crest to the horizontal line of the lowest point of the maxillary sinus in order to calculate the area within the maxillary sinus (yellow area). The midpoint, $\:{P}_{mid}$($\:{P}_{mid}x$, $\:{P}_{mid}y$), of the line connecting the two green-marked points on the alveolar crest is extended 10 mm upward along the y-axis, resulting in a new point $\:{P}_{new}$(༈$\:{P}_{mid}x$, $\:{P}_{mid}y-10\times\:spacing$), where “spacing” refers to the pixel-to-millimeter ratio. A horizontal line is then drawn from this new point, dividing the maxillary sinus mask into two regions. The lower region represents the internal maxillary sinus area, denoted as A5.

### Statistical analysis

This study quantitatively evaluated the consistency between AI segmentation and expert manual annotations using IoU, AP, AR and Euclidean distance. The median, IQR, and 95%CI described the distribution of IoU. Other consistency indicators were described by mean ± standard deviation. Bland-Altman analysis was used to evaluate the consistency between AI automatic segmentation and expert manual segmentation in A1-A5 measurements. All statistical analyses were performed using SPSS Statistics 24.0 (SPSS Inc., Chicago, IL, USA).

## Results

### Maxillary sinus segmentation

Maxillary sinus segmentation achieved a median IoU of 0.945 (IQR: 0.934–0.951, 95%CI: 0.935–0.941). This high IoU value reflects the model’s ability to generate accurate and consistent segmentation results, effectively delineating the boundaries of the target region with minimal error.

### PSAA segmentation

PSAA segmentation achieved a median IoU of 0.991 (IQR:0.982–1.000, 95%CI: 0.948–0.974).

- AR@[IoU = 0.5]

The model successfully identified 100% of PSAA in the test dataset, as shown by its recall of 0.937 ± 0.024 at an IoU threshold of 0.5. The model’s high recall score highlights its sensitivity and capacity to reduce false negatives.

- AP@[IoU=0.5]

The model attained an average precision of 0.902 ± 0.023 at the same IoU threshold, demonstrating its strong overall accuracy in PSAA detection. This result reflects the model’s capability to reliably identify true positives while effectively minimizing false positives, highlighting its precision and robustness.

### Alveolar ridge points detection

**Fig. 4:**
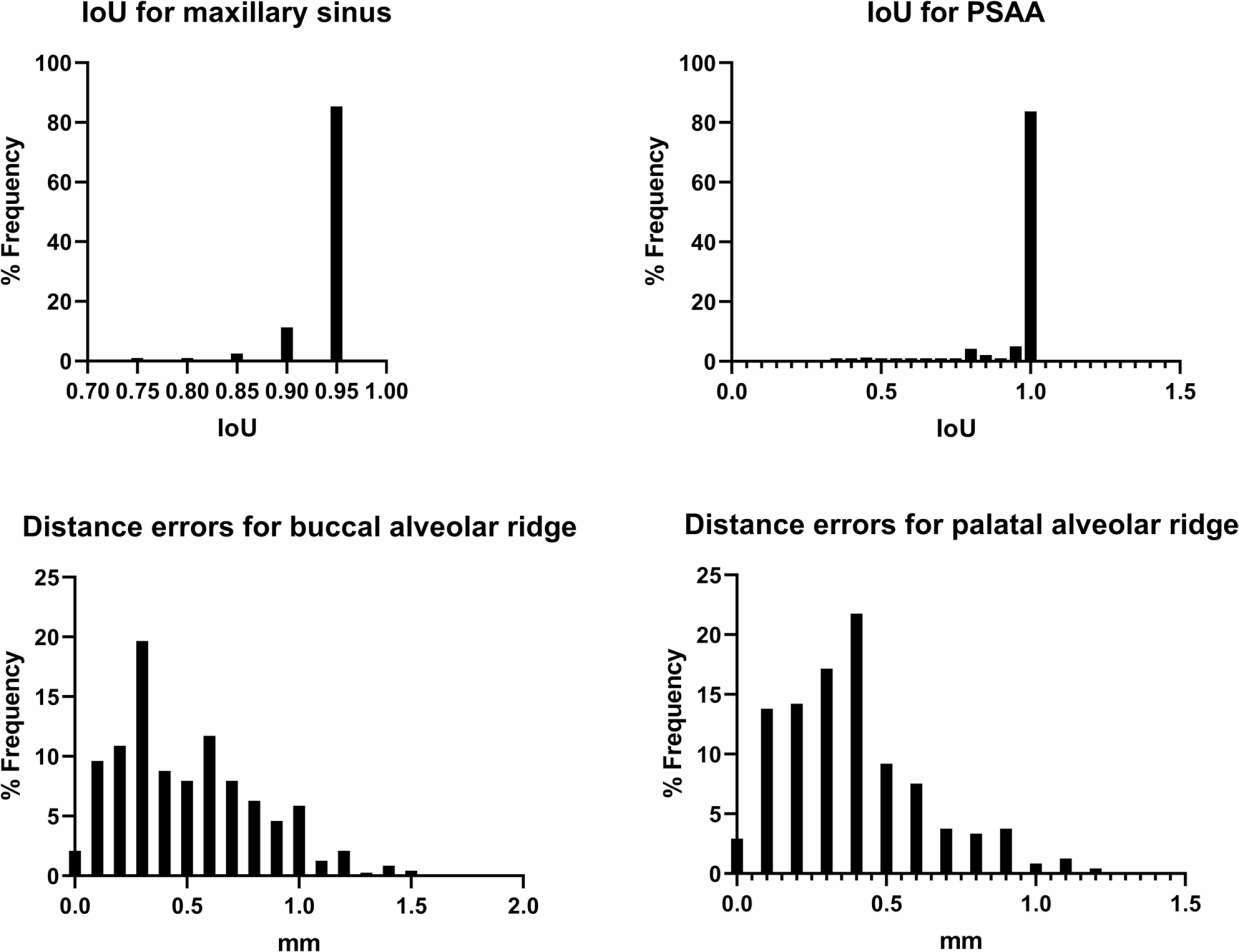
Distribution characteristics of IoU in maxillary sinus and PSAA segmentation. Distribution characteristics of segmentation errors in alveolar ridge points

For alveolar ridge point detection, the Euclidean distance errors between the predicted and ground truth points were 0.50 ± 0.31 mm and 0.38 ± 0.24 mm for the two key points, respectively. The two key points were the alveolar crest buccal turning point and the palatal turning point. The two points were able to assist in the assessment of the implant’s available bone width and bone height, as well as in designing the location of the upper edge of the lateral wall bony window. These small errors suggest that the model achieves high precision in locating alveolar ridge points, with minimal deviation from the annotated positions (as shown in Fig. 4).

### Consistency verification between manual annotations and AI prediction results

**Fig. 5:**
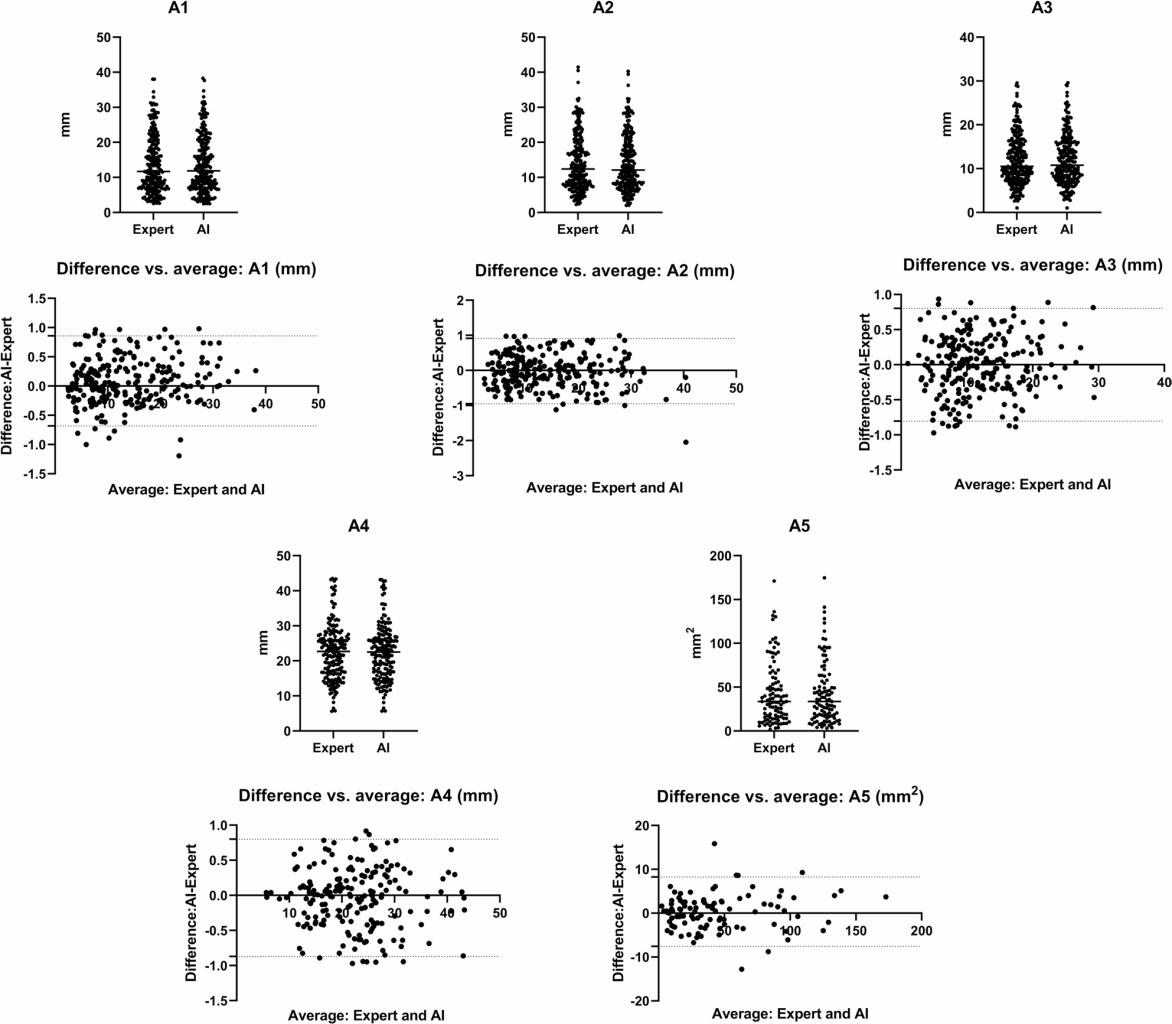
Distribution characteristics of data and errors for A1-A5

The test dataset was used to assess the agreement between manual annotations and automated model predictions. The manually annotated and AI-predicted results for the alveolar crest, the maxillary sinus area, and the PSAA bounding box were used to calculate metrics A1 through A5. Table 1 showed the numerical findings of these computations and the prediction error between manual annotations and automated predictions for each data point. As shown in Figs. 5 and 95% of distance error for A1-A4 were less than 1 mm. 95% of area error for A5 were less than 10 mm^2^.

**Table 1:** A1-A5 related data annotated manually and predicted by AI

Calculations	Mean Expert (mm unit)	Mean AI(mm unit)	Mean ± SD AI-Expert (mm unit)
A1	14.5062	13.8657	0.3102 ± 0.2558
A2	13.8667	14.1600	0.3728 ± 0.2935
A3	11.2590	12.0333	0.3292 ± 0.2431
A4	22.5688	22.4726	0.2665 ± 0.2723
A5	42.9885 mm^2^	43.3473 mm^2^	1.2870 ± 2.2848 mm^2^

## Discussion

CBCT has become an indispensable tool in dentomaxillofacial radiology, offering comprehensive volumetric data of teeth and surrounding alveolar bone with higher quality, lower radiation dose, and faster acquisition times [8]. Despite these advantages, subjective interpretation of CBCT scans can lead to considerable inter- and intra-observer variability, particularly among less experienced clinicians [9]. This highlights the critical need for automated methods to enhance the objectivity, reproducibility, and efficiency of anatomical structure identification and measurement in CBCT images, thereby reducing workload and aiding clinical decision-making.

Artificial intelligence (AI) has demonstrated significant potential across various dental applications, including diagnosis assistance and treatment planning, particularly within dentomaxillofacial radiology [10]. Prior AI research in CBCT has focused on automating tasks such as detecting periapical lesions [11], identifying specific root canal morphology [12], diagnosing root fractures [13], assisting implant planning by assessing alveolar bone volume and detecting the inferior alveolar nerve canal, identifying intraosseous lesions and temporomandibular disorders, and segmenting the mandible [14] or individual teeth, as well as aiding in cephalometric analysis and detecting specific anatomical variants like mesiodens.

Regarding maxillary sinus analysis, several studies have explored AI-driven segmentation. Notably, approaches based on the nnU-Net architecture have shown promising results in segmenting the maxillary sinus in CBCT images [15], achieving high performance metrics, including an F1 score, accuracy, sensitivity, and precision of 0.96, and an IoU of 0.93 in one reported case [16]. While sinus segmentation has received considerable attention, automated detection or segmentation of the PSAA using AI in CBCT has been less explored [17]. Similarly, while some studies have addressed automated segmentation of the alveolar bone [18], research specifically focusing on precise identification and localization of the alveolar ridge apex points for quantitative measurement is also limited.

Addressing these gaps, this study proposes and evaluates an integrated network leveraging an enhanced YOLOv11 framework for the simultaneous tasks of maxillary sinus segmentation, PSAA detection, and alveolar ridge apex identification. The results demonstrate the model’s high performance across these tasks. Specifically, the network achieved a median IoU of 0.945 (IQR: 0.934–0.951, 95%CI: 0.935–0.941) for maxillary sinus segmentation, indicating highly accurate and consistent boundary delineation. Previous studies have shown that the maxillary sinus in CBCT was automatically segmented based on the nnU-Netv2 AI model and the IoU value reached 0.93 [16]. For PSAA detection, the model successfully identified 0.937 ± 0.024 of arteries in the test set at an IoU threshold of 0.5, achieving the accuracy of 0.902 ± 0.023 at the same threshold, demonstrating reliable detection with a low false negative rate. AI tools can significantly improve the detection rate of PSAA [17], but different systems differ in the accuracy of PSAA recognition [19]. This was an inspiring result that PSAA segmentation achieved a median IoU of 0.991 (IQR:0.982–1.000, 95%CI: 0.948–0.974). Alveolar ridge apex identification also showed high precision, with mean Euclidean distance errors of 0.50 ± 0.31 mm and 0.38 ± 0.24 mm for the two key points, signifying minimal deviation from manual annotations. In contrast with previous research results, it can be seen that the model still exhibits satisfactory positioning accuracy and robust multitasking capability while performing three tasks: maxillary sinus segmentation, PSAA segmentation and alveolar crest recognition.

In this study, we adopted an IoU threshold of 0.5 to evaluate detection performance. This threshold is widely used in both general object detection benchmarks (e.g.,Pattern Analysis, Statistical Modelling and Computational Learning Visual Object Classes, PASCAL VOC) and medical image analysis studies, as it offers a reasonable balance between localization accuracy and tolerance to inter-observer variability. In clinical practice, slight differences in lesion boundary annotation are common among radiologists, and an IoU of 0.5 ensures that the detected region sufficiently overlaps with the ground truth to be clinically meaningful. While lowering the IoU threshold would increase recall by accepting more partially overlapping detections as true positives, it would also compromise precision, leading to a higher number of false positives. Since our primary objective was to assess whether the model can reliably localize lesions rather than merely approximate their presence, we considered IoU = 0.5 the most appropriate trade-off. Future work may further explore multi-threshold evaluations (e.g., mean AP across multiple IoU levels) to provide a more comprehensive understanding of model performance.

**Fig. 6:**
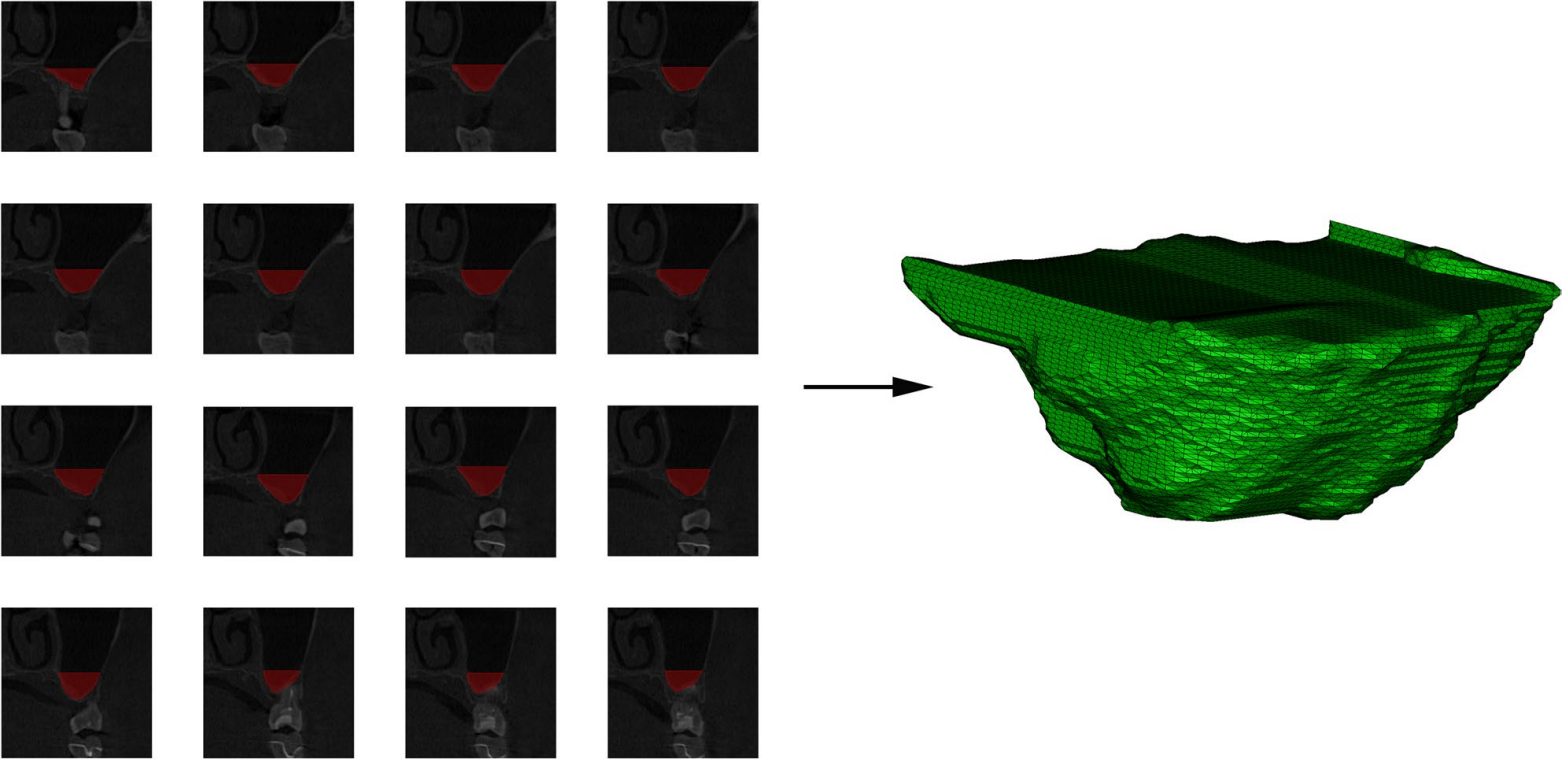
Visualization of predicting bone graft volume using A5’s two-dimensional data

A key contribution of this study lies not only in the high-precision multitasking system but also in integrating these outputs to automatically calculate clinically relevant parameters for implant surgery design. Unlike many previous studies limited primarily to segmentation, our framework computes five specific parameters (A1-A5) directly applicable to pre-operative planning. Parameters A1-A3 quantify available bone height and width based on the alveolar ridge apex points, directly aiding in determining appropriate implant dimensions [20]. Parameter A4 measures the distance from the PSAA to the buccal alveolar crest point, providing crucial spatial information for designing the lateral bony window to avoid vascular injury [21]. 95% of distance error for A1-A4 were less than 1 mm. The surgeon evaluates the implant available bone height by A1 and A2 and the implant available bone width by A3, thus assisting the preoperative design, selecting the parameters of the implant (diameter and length).The surgeon designed the upper edge position of the lateral wall bone window with the aid of A4, avoiding injury to the PSAA and thus avoiding the risk of intraoperative bleeding. Parameter A5, currently in the exploratory stage, aims to estimate the volume of the sinus region targeted for elevation, offering a reference for bone graft material quantity in future clinical applications based on serial image analysis (as shown in Fig. 6). Especially for cases where the vertical bone mass in the maxillary posterior region is severely insufficient, requiring a large amount of bone grafting in one stage and delayed implantation, accurately estimating the volume of bone graft will help improve surgical efficiency and ensure postoperative sinus floor elevation.This integrated approach to automated measurement based on identified anatomical landmarks represents a significant advancement in translating AI capabilities into directly actionable clinical data, potentially improving diagnostic accuracy and substantially reducing the time and cost associated with manual planning.

The successful application of the enhanced YOLOv11 framework to this multi-task problem in complex CBCT images underscores its reliability as an intelligent assistant tool in dental radiology. However, the present study had limitations. First, the data were limited to 2D sections rather than full 3D CBCT volumes, which may underestimate the true complexity of maxillary sinus and PSAA pathways. Secondly, the clinically relevant parameter A5 (estimation of graft volume) was still in the exploratory phase and may not reflect accurate volume assessment in practical applications. In response to the above two shortcomings, we plan to optimize the data in the future on the basis of this study and analyze the complete 3D CBCT volume files, which can more realistically reflect the complexity of the maxillary sinus and PSAA pathways. The volume of the maxillary sinus endograft can be more accurately estimated. In addition, the present study data were all from a single imaging device of a single center, which may limit the universality to other environments and devices. So in the future we will obtain datasets from different devices of multicenter, thus enhancing the commonality and clinical applicability of the framework.

## Conclusions

The AI model based on the enhanced YOLOv11 framework developed in this study demonstrates high performance in automatically identifying and segmenting key anatomical structures related to maxillary sinus elevation (maxillary sinus, PSAA, and alveolar crest). By automatically calculating critical parameters (available bone height-A1 and A2, available bone width-A3, distance from buccal alveolar ridge crest to PSAA-A4) for implant surgery design based on these structures, the model effectively supports clinicians in making informed decisions and planning surgical procedures.

## Supplementary Information

Additional file 1: Raw data of IoU in three anatomical structure (MS,PSAA,ARP) image segmentation using artificial intelligence and expert annotated.

## Data Availability

The datasets supporting the conclusions of this article are included in Additional file 1.

## Abbreviations

PSAA: Posterior superior alveolar artery
CBCT: Cone-beam computed tomography
CNNs: Convolutional neural networks
IoU: Intersection over union
CSP: Cross-stage partial
CSPSA: Cross-stage partial spatial attention
PSA: Position-sensitive attention
AP: Average precision
AR: Average recall
TP: True positive
GT: Ground truth
FP: False positive
FN: False negative
